# The role of personality functioning and childhood trauma in patients in opioid substitution treatment

**DOI:** 10.3389/fpsyt.2025.1584143

**Published:** 2025-06-18

**Authors:** Laura Waschulin, Stefan Hofner, Daniela Bildstein-Ebner, Jürgen Fuchshuber, Susanne Hörz-Sagstetter, Klemens Michlmayr, Ludwig Ohse, Karel D. Riegel, Victor Blüml

**Affiliations:** ^1^ Department of Counselling, Support, Care and Treatment, Suchthilfe Wien gGmbH, Vienna, Austria; ^2^ Department of Psychoanalysis and Psychotherapy, Medical University of Vienna, Vienna, Austria; ^3^ Comprehensive Center for Clinical Neurosciences and Mental Health, Medical University of Vienna, Vienna, Austria; ^4^ Center for Integrative Addiction Research (CIAR), Grüner Kreis Society, Vienna, Austria; ^5^ Department of Clinical Psychology and Psychotherapy, Psychologische Hochschule Berlin, Berlin, Germany; ^6^ Department of Psychology, MSB Medical School Berlin, Berlin, Germany; ^7^ Department of Addictology, First Faculty of Medicine, Charles University and General University Hospital in Prague, Prague, Czechia

**Keywords:** personality functioning, substance use disorders, opioid use disorders, injecting drug use, childhood trauma

## Abstract

**Background:**

Personality pathology and childhood trauma are known to be associated with substance use disorders (SUDs) in general and opioid use disorders (OUDs) in particular but the complex relationship is only partially understood. Investigating personality functioning in patients with OUD is crucial for gaining a deeper understanding of the emergence and course of illness as well as for planning appropriate treatment strategies.

**Aims:**

To empirically investigate personality functioning in a sample of patients in opioid substitution treatment and to examine the associations between personality functioning, injecting drug use (IDU) and childhood trauma.

**Methods:**

In a cross-sectional design, 31 patients with OUDs currently in an opioid substitution treatment program were assessed with the revised Structured Interview for Personality Organization, the Structured Clinical Interview for DSM-5, the Addiction Severity Index – Lite and the Childhood Trauma Questionnaire. The sample consisted of 80.6% male and 19.4% female patients.

**Results:**

The large majority (93.5%) of participants were diagnosed with severe impairment of personality functioning. Impaired personality functioning and higher rates of reported childhood trauma were associated with a younger age of onset of IDU and a greater number of years of IDU. Level of personality functioning showed a stronger statistical association with both IDU and the number of diagnosed personality disorders than reported childhood trauma.

**Conclusions:**

OUDs are associated with severely impaired personality functioning. Assessment of personality functioning can provide important information for treatment strategies in addition to categorical psychiatric diagnoses and trauma history.

## 1 Introduction

Opioid use disorders (OUDs) are serious, often chronic mental disorders characterized by problematic opioid use leading to significant impairment, distress, high mortality risk and substantial impacts on individual health and national healthcare systems (1–3).

In many cases, OUDs are connected to polysubstance use and injecting drug use (IDU) (2, 4). The complex etiology of substance use disorders (SUDs) include biological, genetic, sociocultural and psychological factors (5, 6).

Among the psychological factors, early adverse experiences, particularly childhood trauma, have been increasingly recognized as a critical factor in the development of severe mental disorders, including SUDs (7).

Patients with SUDs frequently describe experiences of severe childhood traumatization (8) and for OUDs, research suggests that more severe childhood trauma is associated with a higher risk of earlier onset of opioid use and IDU (9–12).

In this context, personality pathology has emerged as key psychological factor in SUDs, with growing evidence suggesting an association between certain personality traits and disorders and the occurrence and persistence of substance use disorders (11, 13–17).

Research on co-morbidity of SUDs and other mental disorders has shown elevated prevalence rates of Antisocial and Borderline Personality Disorder (PD) among OUD patients (11, 13, 14). Furthermore, studies examining personality traits in this population revealed higher scores in facets of Neuroticism and lower scores in facets of Conscientiousness, Extraversion, and Agreeableness (15, 16).

Against this background, the concept of “personality functioning” (PF) has gained increasing relevance as a dimensional approach to understanding personality-related vulnerability in psychopathology in general (18, 19), also offering a more nuanced framework for assessing the underlying psychological mechanism in patients with SUDs.

The importance of this concept was underscored by the introduction of the Alternative Model for Personality Disorders in DSM-5 and the revised PD classification in ICD-11, both of which define disturbances in self (identity and self-direction) and interpersonal (intimacy and empathy) functioning as the core features of personality pathology (18, 19).

These models of PF converge with long-standing psychodynamic conceptualizations (20) as in the object relations model developed by Kernberg and colleagues.

Kernberg’s model of personality organization (= functioning) offers a developmentally informed psychodynamic framework for understanding manifestations of personality pathology, particularly in light auf early traumatic experiences. Central to this model is the notion that early relational experiences play a critical role in the internalization of “object relations” and the organization of the self. When early attachment relationships – particularly those involving neglectful, abusive or in other ways traumatic experiences – fail to support the integrations of intense affective experiences, individuals may rely on “primitive” defense mechanisms, such as splitting, leading to impairments in identity integration (identity diffusion), in the perception of the self and others and consequently to difficulties in building healthy relationships and deficits in affect regulation. Kernberg’s theory provides an in-depth understanding of how early trauma and relational disruptions can shape patterns of impaired psychological functioning (21, 22).

The model comprises three basic levels of PF: neurotic, borderline, and psychotic personality organization (23, 24). These levels of personality organization (i.e., PF) are distinguished by differences in identity integration, maturity of defense mechanisms, the capacity for reality testing, and the integration of aggression and moral values. A neurotic level of PF is defined by an integrated identity, relatively mature defense mechanisms (e.g. anticipation), and intact reality testing. Borderline personality organization is characterized by an unintegrated identity (identity diffusion) and the use of primitive defense mechanisms (mainly splitting and projective identification) with intact capacity for reality testing. Based on Kernberg’s model of PF, the “Structured Interview for Personality Organization” (STIPO) was developed, a semi-structured Interview allowing an in-depth assessment of personality pathology (25, 26). A revised and shortened version, the STIPO-R, was subsequently introduced (26). Studies investigating personality pathology among opioid and polysubstance use samples found severe impairments across domains of PF [27, 28, 29). In a study by Fuchshuber et al. (30) deficits in PF were found to mediate the relationship between childhood trauma and addictive behaviors. Moreover, a greater number of comorbid PDs was found to be associated with more severe impairment in PF (9, 29, 31).

The concept of PF provides a scientifically grounded and clinically meaningful framework for examining personality pathology in patients with SUDs and related behaviors (e.g., IDU). It allows for a more comprehensive assessment of psychological functioning that extends beyond descriptive diagnoses and reports of childhood trauma.

## 2 Aims

The main aim of this study is to empirically investigate PF using the STIPO-R in individuals with OUDs currently in a substitution program and to examine the associations between PF, IDU and childhood trauma. We expected significant correlations between all STIPO-R domains, childhood trauma severity and age of the first IDU, as well as number of years of IDU. Moreover, we investigated the extent to which deficits in PF and childhood trauma are independently associated with IDU and with the number of diagnosed PDs via hierarchical multiple regression analysis.

## 3 Methods

The study employed a cross-sectional design. The study project was approved by the Ethics Commission of the Medical University of Vienna. Written informed consent was obtained by all participants.

All interviews were conducted by certified psychotherapists or psychotherapists in advanced stages of psychotherapy training, who received specific training in the administration of each interview. Prior to the beginning of data collection, each interviewer completed a supervised training case involving the full interview process. The results and the procedure were then reviewed and discussed within the research group to ensure consistency in administration and standardized application of all instruments.

The interview process lasted 4 to 5 hours and was divided into 2 to 3 sessions, depending on the participant’s preferences. Breaks were provided whenever needed.

### 3.1 Participants

31 patients in opioid substitution treatment were recruited at *“Suchthilfe Wien”* in Vienna. Participants had to meet the following inclusion criteria: over 18 years old, DSM-5 diagnosis of OUD, sufficient German language skills, and cognitive ability to understand the interviews and questionnaires. Patients with psychotic disorders, current intoxication, or significant cognitive impairment were excluded. Psychotic disorders were assessed using the SCID-5. Acute intoxication was evaluated through conversation and clinical observation at the start of the interview. If intoxication was evident, the interview session was rescheduled or terminated to ensure valid participation. Cognitive impairment was informally screened during consent and early interview stages, with difficulties in understanding or engagement indicating possible impairment. Given the sample’s characteristics (OUDs with frequent polysubstance use), some residual substance effects were expected. Exclusion criteria focused pragmatically on acute impairment that would compromise ethical and valid participation.

Sampling followed a referral-based approach: social workers at *Suchthilfe Wien* were informed about the study and referred patients who appeared to meet the inclusion criteria. A total of 68 individuals were referred and initially agreed to participate in the study. Of these, 30 participants (44.12%) fully completed both the interviews and questionnaires, while one participant (1.47%) completed the interview but not the questionnaires. 15 individuals (22.06%) either lost interest in participation or were unavailable, for example due to hospitalization. 8 participants (11.76%) did not appear for their first scheduled appointment. In ten cases (14.71%), participants did not return after their first appointment and dropped out of the interview process. Additionally, in four instances (5.88%), interviewers determined that the inclusion criteria were not actually met – due to reasons such as active psychosis, not being enrolled in an opioid substitution program, intoxication, or being too adversely affected by the interview process. Demographic data were collected using a brief, self-constructed questionnaire.

Each participant received a 50 Euro voucher as compensation, balancing acknowledgment of their time without exerting undue influence on their decision to participate.

### 3.2 Measures

#### 3.2.1 Structured interview for personality organization-revised

The Structured Interview for Personality Organization (STIPO) is a semi-structured interview assessing personality functioning based on Kernberg’s object relations model (25, 26). The most recent version, the revised STIPO (STIPO-R), consists of 55 items and assesses the following domains and sub-domains: 1. Identity: 1.A. Capacity to invest, 1.B. Sense of self - Coherence and continuity, 1.C. Representation of others; 2. Object relations: 2.A. Interpersonal relationships, 2.B. Intimate relationships and sexuality, 2.C. Internal working model of relationships, 3. Defense: 3.A. Primitive defenses, 3.B. Higher-level defenses; 5. Aggression: 5.A. Self-directed aggression, 5.B. Other-directed aggression 6. Moral values. It also includes a rating of narcissism. The single-item rating is made by the interviewer on a three-point scale with operationalized descriptions for each rating. For each (sub)dimension a clinical rating can be made on a 1-to-5 scale, allowing for a clinical assessment based on operationalizations of the domains. From the clinical ratings, an overall level of personality organization can be determined. Six different levels of personality organization are provided for the overall rating, ranging from a normal level to severely impaired PF: (1) Normal, (2) Neurotic 1, (3) Neurotic 2, (4) Borderline 1, (5) Borderline 2, and (6) Borderline 3 (26). For this study, the 1–5 clinician ratings across the six main domains, along with the overall rating of level of PF (scale range: 1–6), were used for statistical analysis.

Satisfactory reliability and validity have been demonstrated for the STIPO (32–34). For this study, the intraclass correlation coefficient (ICC) among the interviewers for the overall STIPO level was.90.

#### 3.2.2 Structured clinical interview for DSM-V

The Structured Clinical Interview for DSM-5 (SCID-5) is the official instrument for the diagnosis of psychiatric disorders according to DSM-5. The semi-structured interview contains questions addressing every single diagnostic criterion of the psychiatric disorders of the DSM-5 (35, 36). For the present study, the German versions of SCID-5-PD and SCID-5-CV were used (37, 38). The variable ‘number of PDs’ was obtained using the SCID-5-PD by summing the categorical PD diagnoses for each participant.

#### 3.2.3 Addiction severity index-lite

The Addiction Severity Index-Lite (ASI-Lite) is a shortened version of the Addiction Severity Index (ASI), a semi-structured interview that assesses substance use-related behaviors and problems over a lifetime and the past 30 days (39). The English ASI-Lite has psychometric properties similar to the original ASI (40). A slightly abbreviated German version was used, including an added section on IDU from the original ASI (41). Participants’ reports on IDU were utilized for statistical analyses.

#### 3.2.4 Childhood trauma questionnaire

The Childhood Trauma Questionnaire (CTQ), in its short version (42), is the most widely used self-report instrument for assessing childhood trauma, consisting of 28 items across five scales: Emotional Abuse, Physical Abuse, Sexual Abuse, Emotional Neglect, and Physical Neglect. The German translation has validated psychometric properties (43). Each subscale score ranges from 5 to 25, based on five items rated on a 5-point scale Likert scale. The total score ranges from 25 to 125, summing the five abuse/neglect subscales (minimization items are excluded from the total score).

Internal consistency was excellent for the CTQ total score (Cronbach’s α = .95), and excellent to good for the Emotional Abuse (α = .90), Physical Abuse (α = .98), Sexual Abuse (α = .89) and Emotional Neglect (α = 0.90) subscales. The Physical Neglect subscale showed lower internal consistency (α = .50). This finding is consistent with previous research indicating that the Physical Neglect subscale tends to show lower internal consistency compared to the other subscales (44, 45).

### 3.3 Statistics

Spearman-Rho-Correlation analyses were conducted to explore the relationship between PF and childhood trauma, PF and IDU and childhood trauma and substance use (one-sided). Multiple hierarchical regression analyses were conducted to examine the predictive value of PF and the CTQ score as independent variables for years of IDU, age of first IDU and total number of PD diagnoses. Multicollinearity was tested using Variance Inflation Factors (VIF). Independence of errors was assessed with the Durbin-Watson-test. All assumptions were met. Analyses were carried out with SPSS 27.

## 4 Results

### 4.1 Sample characteristics

The sample consisted of 31 participants aged between 29 and 66 years (mean = 42.84, SD = 10.05). 80.6% of participants identified as male, 19.4% as female. 90.3% of participants were born in Austria. Most of the sample reported being unemployed (87.1%) and not having a high school diploma (93.5%). More than half of the participants reported being single (64.5%) or divorced (16.1%) at the time of interviewing, 35.5% reported having children.

### 4.2 SCID-5 diagnoses

All participants fulfilled criteria for OUD, and 87.10% of participants were diagnosed with one or more PDs. A detailed list of all SCID-5 diagnoses is shown in **Table 1**.

**Table 1:** SCID-diagnoses.

SCID-5 Diagnoses	n	%
Substance Use Disorders		
Opioids (total)	31	100
* severe*	*31*	*100*
Alcohol (total)	7	22.58
* mild*	*2*	*6.45*
* moderate*	*3*	*9.68*
* severe*	*2*	*6.45*
Sedatives, Tranquilizers, Hypnotics (total)	4	12.90
* severe*	*4*	*12.90*
Cannabis (total)	12	38.71
* mild*	*3*	*9.68*
* moderate*	*5*	*16.13*
* severe*	*4*	*12.90*
Stimulants (total)	10	32.26
* mild*	*2*	*6.45*
* moderate*	*7*	*22.58*
* severe*	*1*	*3.23*
Hallucinogens (total)	1	3.23
* severe*	*1*	*3.23*
Depressive Disorders & Bipolar and Related Disorders		
Major Depressive Disorder (MDD)	6	19.35
MDD partial remission	4	12.90
MDD full remission	6	19.35
Bipolar 1 Disorder (current episode: hypomanic)	1	3.23
Anxiety Disorders & Trauma- and Stressor-Related Disorders		
Panic Disorder	1	3.23
Social Anxiety Disorder	3	9.68
Generalized Anxiety Disorder	5	16.13
Posttraumatic Stress Disorder	13	41.94
* current*	*9*	*29.03*
* Past*	*4*	*12.90*
Personality Disorders		
Avoidant	4	12.90
Dependent	*1*	3.23
Obsessive-Compulsive	*1*	3.23
Paranoid	3	9.68
Schizoid	3	9.68
Narcissistic	1	3.23
Borderline	8	25.81
Antisocial	9	29.03
Other specified PD	11	35.48
* Of those: with antisocial traits*	*8*	*25.81*
Total number of PD diagnoses		
0	4	12.90
1	16	51.61
2	8	25.81
3	3	9.68

### 4.3 Substance use

All participants were in opioid substitution treatment. 16.13% of patients were treated with levomethadone, 3.23% with methadone, and 80.65% with extended-release morphine. There is a relatively high prevalence of polysubstance use in the study sample (cf. **Table 1**). On average, participants used more than one substance per day over a period of 14.58 years (SD = 11.10), and on 17.77 days out of the last 30 days (SD = 13.24). Substances most used in the last 30 days were sedatives/tranquilizers/hypnotics (mean = 19.28 days, SD = 14.60), followed by cannabinoids (mean = 13.35 days, SD = 13.26). The mean age of the first consumption of heroin was 19.17 (SD = 4.38).

Concerning the issue of IDU (referring to the intravenous consumption of any substance, including substitution medications), only 4 participants (12.90%) reported having never injected any substance, the majority (n = 27, 87.10%) confirmed engaging in IDU. The mean age of the first drug injection was 21.67 (SD = 6.13), with an average duration of IDU of 13.90 years (SD = 11.23). Over the last 30 days, participants reported an average of 11.85 days with IDU (SD = 2.52).

### 4.4 Level of PF

**Figure 1:**
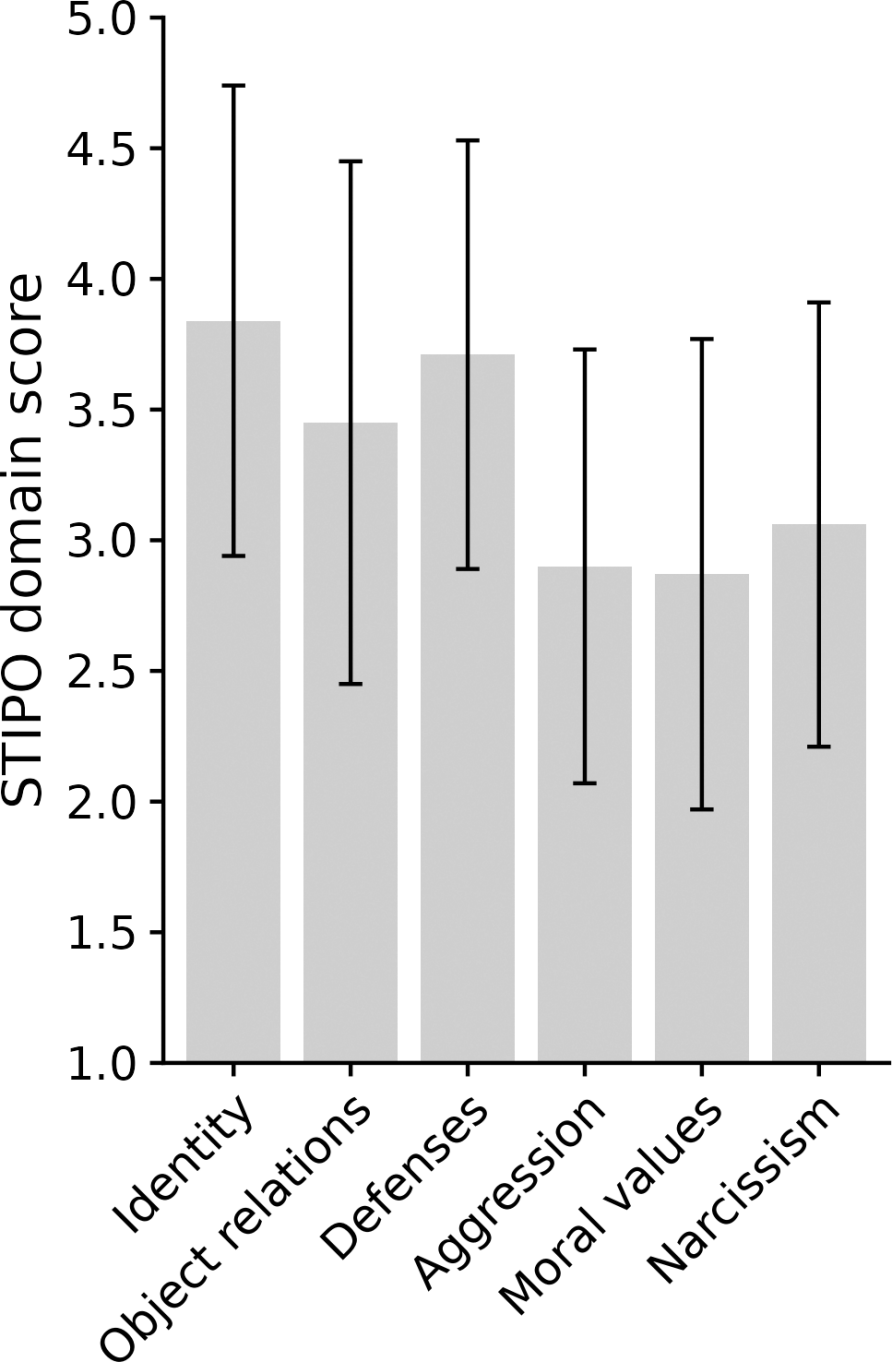
Mean values for the STIPO-R domains (scale-range = 1-5) with standard deviations shown as error bars with 95 % confidence intervals are displayed. Higher values indicate higher levels of pathology.

Most participants were diagnosed with a borderline level of PF (93.50%): 32.30% with “Borderline 1”, 35.50% with “Borderline 2” and 25.80% with “Borderline 3”. Only two participants (6.50%) were diagnosed with “Neurotic 2”. Mean values for the STIPO-R domains are shown in **Figure 1**.

### 4.5 Childhood trauma

Results concerning (remembered) childhood traumatization are shown in **Table 2**. Scores in the different subdomains are grouped into four categories: no traumatization, low to moderate, moderate to severe, and severe to extreme traumatization (42).

**Table 2:** CTQ subdomain scores and distribution across CTQ categories for trauma severity.

CTQ Scale		None	Low - moderate	Moderate-severe	Severe-extreme
	M *(SD)*	n (%)	n (%)	n (%)	n (%)
Emotional Abuse	13.27 (6.23)	11 (35.48)	3 (9.67)	5 (16.13)	11 (35.48)
Physical Abuse	10.67 (7.39)	18 (58.10)	0 (0)	1 (3.23)	11 (35.48)
Sexual Abuse	7.13 (3.50)	20 (64.52)	1 (3.23)	5 (16.13)	4 (12.90)
Emotional Neglect	13.97 (5.47)	7 (22.58)	10 (32.33)	4 (12.90)	9 (29.03)
Physical Neglect	10.37 (3.71)	8 (25.81)	5 (16.13)	9 (29.03)	8 (25.81)
CTQ Total Score	55.40 (22.17)				

Missing: n =1.

When summarizing data categorized “low/moderate to extreme” as positive exposure to childhood traumatization, 61.28% of the participants report emotional abuse, 38.71% physical abuse, 32.26% sexual abuse, 74.26% emotional neglect and 70.97% physical neglect.

### 4.6 The relationship between levels of PF and childhood trauma

The overall level of PF (higher ratings indicating higher levels of pathology) showed moderate to strong significant positive correlations with the CTQ total score and almost all subdomains of the CTQ, except for “emotional neglect” (detailed results are given in **Table 3**).

**Table 3:** Spearman-Rho correlations between STIPO-R domains and CTQ scales.

	Emotional abuse	Physical abuse	Sexual abuse	Emotional neglect	Physical neglect	CTQ total score
Level of PF	.41^*^	.54^**^	.34^*^	.19	.47^**^	.41^*^
Identity	.42^*^	.51^**^	.32^*^	.22	.48^**^	.42^*^
Object Relations	.41^*^	.40^*^	.37^*^	.20	.41^*^	.40^*^
Defenses	.36^*^	.42^*^	.29	.16	.48^**^	.38^*^
Aggression	.42^*^	.48^**^	.25	.15	.25	.34^*^
Moral Values	.28	.47^**^	.40^*^	-.10	.17	.24
Narcissism	.37^*^	.43^**^	.30	.03	.31^*^	.32^*^

** ≤ 0.01, * ≤ 0.05 (one-sided).

### 4.7 The relationship between levels of PF and IDU

The overall Level of PF (higher values indicating higher levels of pathology) was significantly negatively correlated with “age at first IDU” (r = -.52, p <.01) and significantly positively correlated with “years of IDU” (r = .49, p <.01).

Regarding the STIPO-R dimensions, results revealed significant negative correlations between “age at first IDU” and increased pathology in the domain of “identity” (r = -.48, p <.05) and between “age at first IDU” and deficits in the domain of “moral values” (r = -.47, p <.05).

“Years of IDU” showed a significant positive correlation with higher levels of pathology in the following domains: “identity” (r = .45, p <.05), “defenses” (r = .40, p <.05) “aggression” (r = .42, p <.05) and “narcissism” (r = .39, p <.05). Correlations between the other STIPO-R domains and the IDU variables were non-significant.

### 4.8 The relationship between childhood trauma and IDU

“Age at first IDU” showed significant negative correlations with the CTQ total score (r = -.36, p <.05), “physical abuse” (r = -.43, p <.05), “sexual abuse” (r = -.43, p <.05), and with “physical neglect” (r = -.35, p <.05).

“Years of IDU” positively correlated with the CTQ total score (r = .33, p <.05), “physical abuse” (r = .43, p <.01) and “emotional abuse” (r = .35, p <.05).

“IDU in the last 30 days” only sowed a positive correlation with “emotional neglect” (r = .37, p <.05). Correlations between the other CTQ scales and the IDU variables were non-significant.

### 4.9 Regression models of “years of IDU” and “age at first IDU”

Two hierarchical multiple regression analyses were conducted to assess the predictive value of PF and CTQ scores for years of IDU and age at first IDU (**Table 4**). In both models, PF proved to be the stronger predictor for the respective IDU variables than childhood trauma.

**Table 4:** Regression analyses summary.

	Years of injecting drug use			Age of first injecting drug use		
Models and predictor variables	β	R^2^	ΔR	β	R^2^	ΔR^2^
*Regression Model 1*		.13*	.13*		.07	.07
CTQ Total Score	.36*			-.26		
*Regression Model 2*		.29*	.16*		.23*	.16*
CTQ Total Score	.17			-.10		
STIPO-R Level of PF	.44*			-.43*		

* ≤ 0.05.

### 4.10 Number of PDs

The total number of PD diagnoses showed a significant positive correlation with level of PF (r = .77, p <.05). In a multiple regression analysis, PF was shown to be a stronger predictor of the number of PD diagnoses than childhood trauma (cf. **Table 5**).

**Table 5:** Regression analysis predicting number of PDs.

Models and predictor variables	B	SE B	β	R^2^	ΔR^2^
Regression Model 1**				.24**	.24**
(Intercept) CTQ total score	.30 .02	.37 .01	.49**		
Regression Model 2**				.59**	.35**
(Intercept) CTQ Total Score STIPO-R Level of PF	-2.03 .01 .61	.56 .01 .13	.20 .66**		

** ≤ 0.01, * ≤ 0.05.

## 5 Discussion

The results of this study demonstrate that IDU is associated with childhood trauma as well as impairments in PF as measured by the STIPO-R.

Consistent with studies assessing PF in SUD, and especially OUD or polysubstance use samples, most of the participants of this study showed moderate to severe deficits in PF (27–29).

Also, high rates of PDs, especially Antisocial PD and Borderline PD, were observed, alongside elevated reports of childhood trauma, consistent with other findings in SUD patients (46). In line with previous research, a greater number of PDs was associated with increased impairment in PF (29, 31).

Results of the present study show that a younger age at the onset of IDU and a greater number of years of IDU are associated with higher rates of reported childhood trauma and with higher levels of personality pathology. However, in multiple regression analyses, PF demonstrated a stronger statistical association with IDU and the number of diagnosed PDs, accounting for a greater proportion of explained variance than the CTQ score. This finding corresponds to results by Fuchshuber et al. which suggested a mediating role of PF (assessed with the IPO) in the association between childhood trauma and addictive behaviors (30).

In the present study, PF was operationalized with the STIPO-R, an interview based on psychoanalytic object relations theory. Following the central premises of this theory, psychological functioning develops in interactions with early “objects”, i.e. significant others.

Traumatic experiences, defined as overwhelming events that disrupt the psyche’s protective barriers and coping mechanisms, can distort internalized images of self and others and lead to maladaptive defense strategies (23, 24, 47). Severe childhood trauma fosters the internalization of dysfunctional object relations, negatively impacting development and resulting in deficits in PF (23, 24, 48). These deficits are associated with the manifestation of symptoms and mental disorders (31, 49–51).

The STIPO-R domains allow for a nuanced understanding of personality pathology in OUDs. Deficits in the domain of “identity” are associated with an unstable and poorly integrated sense of self and significant others and corresponding disturbances in affect regulation. Substance use might help in the regulation of self-perception and self-worth and in dealing with intense and overwhelming emotional states (23, 52, 53). Disturbances in the dimension of “object relations” point to unstable, fragmented, and negative internal representations of others, leading to severe difficulties in building and maintaining relationships (23, 24). Substance use can function as a coping strategy in the face of disappointments stemming from human interactions, defending against unbearable wishes for and fear of dependency. Severe personality pathology is associated with the use of primitive defense mechanisms like denial, splitting, idealization/devaluation, or projective identification (24, 54). Especially the mechanisms of denial of internal and external reality and of splitting have been emphasized in psychodynamic literature on substance use (51, 55). Furthermore, these defense mechanisms can be reinforced by the pharmacological effects of drugs (55). As has been pointed out in the “self-medication-hypothesis”, substance use can be understood as a mean to alleviate painful affective states, providing a sense of control (52). Considering the STIPO-R domain of “aggression”, drug use and its consequences can also be understood as a form of self-destructive behavior, leading to serious health problems and severe self-neglect (23, 56). The relation between substance use and aggression directed towards others is complex: some substances, but also severe craving and withdrawal symptoms can lead to disinhibition and increased display of aggressive behaviors. However, some individuals among SUD samples seem to show a low control of impulses and high readiness for aggressive actions as part of their personality organization (23). These considerations also touch the domain of “moral values” assessed by the STIPO-R. Because many substances are illegal, maintaining a socially accepted drug addiction is impossible, and being labeled a ‘drug addict’ is stigmatized, often leading to a downward social spiral. It seems crucial to not only assess observable behavioral aspects of illicit actions but to obtain information about the underlying dimensions of PF (e.g., information on the ability to reflect on behavior and articulate and tolerate feelings of guilt).

From psychodynamic perspectives, opioid use in particular has been linked to regressive tendencies, such as the desire for a symbiotic state due to unmet basic needs for safety and closeness, and has been discussed as a defense against cruel self-judgment, resulting feelings of guilt and shame, and potential psychotic disintegration (17).

Considering the application of drugs, IDU takes on a special position: due to its considerable risks, it can be viewed as a form of self-destructive behavior, but it can also be understood as serving a very “existential” purpose: like self-harming behaviors such as cutting, it may be used to release unbearable tension, solidify a sense of self and identity and provide a feeling of control and safety (57, 58).

Regarding the age of onset of IDU, it is important to note that distinct etiological pathways may be involved. Adolescence and early adulthood are sensitive neurodevelopmental periods during which vulnerabilities – such as impulsivity, difficulties with executive functioning or emotional dysregulation – can increase risk-taking behaviors, including early substance use (59). These behaviors may disrupt developmental trajectories and contribute to a more severe course of addiction. In contrast, late-onset IDU may be linked to psychosocial stressors or traumatic events in adulthood, that overwhelm an individual’s emotional regulation and coping capacities.

Additionally, while impaired PF may contribute to substance use, it is furthermore important to consider that prolonged opioid use may itself negatively impact personality functioning. As substance use becomes a central aspect of an individual’s life, it can further erode the sense of self, impair emotional regulation and the ability to build and maintain interpersonal relationships and strengthen maladaptive defense mechanisms. These effects may reinforce and interact with pre-existing vulnerabilities and contribute to a cycle of psychological dysfunction and continued substance use.

Lastly, complementing the psychoanalytic framework, neurobiological models offer important insights by highlighting how repeated substance use alters brain neurochemistry and circuits involved in reward, stress and executive control – especially within the mesolimbic dopamine system and extended amygdala (59). These changes impair emotion regulation, impulse control and decision-making while increasing sensitivity to drug-related cues and stress, leading to craving, withdrawal, and compulsive use (59).

Behavioral models of substance use disorders emphasize the role of reinforcement in the development and maintenance of addictive behaviors, focusing on the pleasurable effects of substance use (positive reinforcement) and the relief from distress or withdrawal (negative reinforcement). Over time, these reinforcement processes strengthen substance use patterns, making them increasingly resistant to change and contributing to an erosion of alternative coping strategies (60).

Although psychodynamic, neurobiological and behavioral models offer different perspectives – focusing on internal conflicts, brain circuitry or learned reinforcement patterns – they each capture distinct facets of addiction. Across these models, substance use is understood as serving a crucial function within an individual’s psychological – and neurobiological – systems, often associated with the compensation of deficits in affect regulation, coping or reward processing.

### 5.1 Implications for treatment and prevention strategies

In the treatment of patients with OUDs, both diagnostic assessment and psychotherapeutic interventions should take facets of PF into account. Integrating PF assessment into treatment planning may help identify individuals at higher risk for treatment dropout, poor adherence, or difficulties in establishing therapeutic alliance – factors known to impact treatment outcomes. Moreover, assessing PF can guide clinical decision-making by identifying specific impairments in mental functioning that may serve as targets for therapeutic interventions (61–65). Longitudinal research is needed to further examine the predictive value of PF for various treatment outcomes and to evaluate the efficacy of therapeutic approaches informed by PF assessment.

Given the complex pathogenesis of OUDs, a multiprofessional treatment approach is required, combining psychotherapy, social work and medical care. Since childhood trauma is a major risk for mental health issues, prevention strategies should prioritize not only restricting access to illegal drugs but also fostering supportive social networks and ensuring accessible healthcare for families and children in challenging circumstances.

### 5.2 Limitations

Limitations of this study include its cross-sectional design and the relatively low sample size due to the time-consuming interview process. Childhood trauma was assessed using a self-report questionnaire, which may be subject to various biases (e.g. recall bias, limited insight into one’s own traumatic experiences). Additionally, the sample primarily consisted of male patients, reflecting the common overrepresentation of males in this population (66) preventing the consideration of gender differences.

Another limitation concerns the risk of inflated Type I error due to multiple statistical comparisons. Formal corrections for multiple testing were not applied, a decision based on the exploratory nature of the study and the limited sample size, a consequence of the time-intensive and resource-demanding interview procedure. Applying strict correction methods could have reduced statistical power and potentially obscured meaningful patterns for further research. Nevertheless, the lack of correction increases the likelihood of false-positive findings, and the results should therefore be interpreted with appropriate caution. Replication in larger, more statistically powered samples is needed to confirm the robustness of the observed results.

Finally, it should be emphasized that causality cannot be inferred due to the cross- sectional study design. While significant associations between personality functioning and substance use were observed, the direction of these effects cannot be determined. It seems plausible that impairments in personality functioning contribute to substance use disorder pathology, but also that prolonged substance use may negatively impact personality functioning.

## 6 Conclusion

OUDs are associated with severely impaired PF. A younger age at the onset of IDU and a greater number of years of IDU use are associated with higher levels of personality pathology and reports of childhood trauma. A greater number of PDs was associated with more severe impairment of PF. When compared, PF accounts for a greater proportion of explained variance in IDU and the number of diagnosed PDs than self-reported childhood trauma. Assessment of PF can provide important information for preventive and treatment strategies in addition to categorical psychiatric diagnoses and trauma history anamnesis and enhances our understanding of the complex relationship between substance use, childhood trauma and personality pathology.

## Data Availability

The raw data supporting the conclusions of this article will be made available by the authors, without undue reservation.
